# Staphylococcus aureus and repeat bacteremia in febrile patients as early signs of sternal wound infection after cardiac surgery

**DOI:** 10.1186/1749-8090-9-80

**Published:** 2014-05-08

**Authors:** Teruya Nakamura, Takashi Daimon, Norio Mouri, Hirotada Masuda, Yoshiki Sawa

**Affiliations:** 1 Division of Cardiovascular Surgery, National Hospital Organization Kure Medical Center, Kure, Japan; 2 Department of Biostatistics, Hyogo College of Medicine, Nishinomiya, Japan; 3 Department of Surgery, Osaka University Graduate School of Medicine, Suita, Japan

**Keywords:** Infection, surgical wound, Staphylococcus aureus, Bacteremia, Anti-bacterial agents, Thoracic surgery

## Abstract

**Background:**

Sternal wound infection is a devastating complication of cardiothoracic surgery that carries high postoperative morbidity and mortality rates. We explored whether our current program of extensive bacteriological examination including repeat blood cultures may contribute to the early diagnosis of sternal wound infection.

**Methods:**

We retrospectively analyzed 112 patients who were subjected to our bacteriological examination protocol including within 90 days after cardiothoracic surgery. Univariate and multivariate analyses were made in order to identify risk factors for sternal infection.

**Results:**

The median patient age was 75 years, and 65 patients were male. In 35 cases (31.2%) the blood cultures showed the presence of bacterial infection with the following frequencies: *Staphylococcus aureus*, 18 cases; Coagulase-negative *Staphylococcus*, 7 cases; other organisms, 10 cases. Eleven patients presented repeat bacteremia on at least 2 different occasions. Twenty patients (17.8%) presented sternal wound infections. There was no difference in operative mortality between the patients with and without sternal wound infection. Univariate and multivariate analyses demonstrated that bilateral mammary artery use (OR, 13.68, 95% CI, 1.09-167.36, *p* = 0.043), positive blood culture for *Staphylococcus aureus* (OR, 19.51, 95% CI, 4.46-104.33, *p* < 0.0001), repeat bacteremia (OR, 17.98, 95% CI, 2.51-161.77, *p* = 0.004) were risk factors that were associated for sternal wound infection.

**Conclusion:**

Repeat blood cultures in febrile patients appear to be useful for the early detection of *Staphylococcus aureus* and repeat bacteremia, and these were associated with sternal wound infection. Bilateral internal mammary artery use was another risk factor of sternal wound infection in febrile patients. These factors may identify patients suitable for expeditious radiological examination and aggressive treatments.

## Background

Postoperative sternal wound infection in cardiac surgery is an infrequent complication, with an incidence being 0.25-10% [1,2]. Despite marked progress in precautious modalities, the incidence seems to be unchanged over the past decades [3]. Sternal wound infection generally causes considerably high morbidity and mortality, prolonged hospital stay and increased medical expenditure [1-4]. Although early diagnosis and initiation of surgical exploration are the mainstays of treatment [4-6], it is often difficult to diagnose deep sternal infection in the earlier stages because of delayed interpretation of bacteriological and radiological studies.

In 2006, we implemented an active screening program to analyze the active screening program with extensive bacteriological examination on febrile patients after cardiothoracic surgery, with the aim of early detection and treatment of serious systemic infection. The program consists of preoperative nasal samplings of all patients, and postoperative repeat cultures of blood, urine, sputum and stools for febrile patients. The purpose of the study is to describe how various clinical characteristics and perioperative factors affect early diagnosis of postoperative sternal wound infection, focusing on the significance of routine blood culture protocols in identifying sternal wound infection and prioritizing opportunities for intervention.

## Methods

Our standard bacteriological examination and treatment protocol for postoperative febrile patients commenced in July 2006, and by the end of 2012, 740 patients had undergone cardiothoracic surgery via sternotomy at our institute. Of them, 112 patients who were subjected to bacteriological examination within 90 days of surgery were considered most susceptible to postoperative sternal wound infection, and these patients were included in this study. This represents 15% of all cardiothoracic patients in the same period. Patients who had obvious local signs of wound infection (tenderness, redness and swelling, sternal instability, or purulent discharge from the incision) prior to fever elevation were excluded from the study. The institutional review board (the ethical committee chair: Kiyomi Taniyama, M.D.) approved this study, and patient consent was waived due to the retrospective nature of the study. Reports of all blood cultures drawn in the cardiac surgery unit were obtained from the microbiology laboratory database. All other clinical and laboratory data were reviewed and obtained from the electronic medical records.

Preoperatively, all patients underwent nasal culture to identify carriers of *Staphylococcus aureus*, and intranasal mupirocin was prescribed for all carriers. In the majority of cases the surgeries were performed using cardiopulmonary bypass, while coronary bypass and other procedures had been carried out using an off-pump technique in the remainder of cases. Standard techniques for preparation of the skin and draping were carried out based on the guidelines of the Centers for Disease Control and Prevention (CDC) [7]. A standard regimen of antibiotic prophylaxis consisted of 1 gram of cefazolin 30 minutes before skin incision, 4 hours after the initial dose and every 12 hours thereafter. These prophylactic doses of antibiotic were discontinued on postoperative day 2. In the case of patients with a severe allergy to β-lactam, vancomycin was used. In most cases, chest tubes and temporary pacemaker wires were removed on postoperative day 2. Skin incisions were protected for at least 48 hours, and were inspected daily by cardiothoracic surgeons, and by the institutional wound care team when necessary. The target blood glucose level was below 140 mg/dl, achieved either by continuous or preprandial administration of insulin.

Febrile patients were defined as those with an axillary temperature above 38°C. Our standard protocol of extensive bacteriological examinations was applied to all febrile patients, and consisted of culturing 2 sets of blood, sputum urine and stool samples on 2 consecutive days. Sampling of sputum was performed either by expectoration, sputum induction, or suction via an endotracheal tube using a suction tube if patients are intubated. Urinary samples are usually collected as clean-catch midstream samples. If patients were unable to spontaneously urinate, urine was collected by inserting a urethral catheter. This protocol was applied to all patients within 90 days after surgery. Incisions were explored if there were signs of infection or sternal instability. The wound infections including both the superficial and deep/mediastinal space were defined based on the CDC guidelines [8]. Upon finding signs of sternal wound infection, additional intravenous antibiotic regimens that covered gram-positive bacteria, usually vancomycin or daptomycin [4,5], were resumed. These were either changed or discontinued according to clinical conditions and the culture test results.

Statistical analyses were performed using JMP® 10 (SAS Institute Inc., Cary, NC). Continuous variables are summarized as medians with minimums and maximums, and categorical variables as frequencies with proportions. To explore potential risk factors associated with sternal wound infection, univariate analyses were performed using the Mann–Whitney *U* test for continuous variables, and Fisher’s exact test for categorical variables. The risk factors for sternal wound infection that were found to be statistically significant in univariate analyses were included in multivariate logistic regression analysis. The results are reported as odds ratios (ORs) with 95% confidence intervals (CIs) and *p* values, which were based on likelihood ratio statistic. All statistical tests were two-sided, and *p* values of < 0.05 were considered statistically significant.

## Results

Table 1 shows patient characteristics and perioperative data. The median age was 75 years, with a male to female ratio of 65:47. Thirty six percent of patients were diabetic, 40% of whom were on preoperative insulin therapy. Seven patients were preoperative nasal carriers of *Staphylococcus aureus*. Coronary bypass surgery accounted for 29.5% of the cases, whereas valve repair and/or replacement, and thoracic aorta accounted for 18 cases (16.1%) and 31 cases (27.7%), respectively. The mean and median European system for cardiac operative risk evaluation (EuroSCORE) were 8.7% and 4.5% in this series, respectively. There were 13 operative mortalities (11.6%). About half of the cases had 1 or more episodes of fever elevation for more than 3 days.

**Table 1:** Patient characteristics and univariate analysis of sternal wound infection

**Variable**		**Sternal infection**	**Sternal infection**	
	**All patients**	**Absent**	**Present**	**p value**
	**(n = 112)**	**(n = 92)**	**(n = 20)**	
Age	71.3 ± 11.0	71.9 ± 12.6	72.5 ± 8.7	.543
Male (%)	65 (58.0)	54 (58.7)	11 (55.0)	.806
BMI, kg/m2	22.5 ± 5.0	22.7 ± 5.2	21.3 ± 4.0	.199
Hypertension (%)	85 (75.9)	66 (71.7)	19 (95.0)	.040
Hyperlipidemia (%)	49 (43.8)	38 (41.3)	11 (55.0)	.323
Diabetes (%)	41 (36.7)	31 (33.7)	10 (50)	.204
Insulin use (%)	16 (14.4)	10 (11.0)	6 (30.0)	.040
Renal failure (%)	57 (50.9)	49 (53.3)	8 (40.0)	.330
Chronic lung disease (%)	18 (16.1)	16 (17.4)	2 (10.0)	.521
Steroid use (%)	4 (3.6)	3 (3.3)	1 (5.0)	.550
NYHA class III or IV (%)	47 (42.0)	40 (43.4)	7 (35.0)	.619
LVEF, %	57.7 ± 13.3	58.1 ± 13.0	55.7 ± 14.4	.471
Coronary disease	47 (42.0)	37 (40.2)	10 (50.0)	.461
Pulmonary hypertension	37 (33.3)	30 (32.6)	7 (36.8)	.791
Nasal colonization of SA (%)	7 (6.3)	4 (4.4)	3 (15.0)	.107
EuroSCORE, %	8.7 ± 11.0	8.9 ± 11.5	8.0 ± 8.6	.936
Type of operation				.390
Coronary bypass	33 (29.5)	23 (25.0)	10 (50.0)	
Aoric Valve replacement	16 (14.3)	15 (16.3)	1 (5.0)	
Mitral Valve repair/replacement	18 (16.1)	16 (17.4)	2 (10.0)	
Double Valve	4 (3.6)	3 (3.3)	1 (5.0)	
Triple Valve	8 (7.1)	7 (7.6)	1 (5.0)	
Thoracic aorta	31 (27.7)	26 (28.3)	5 (25.0)	
Others	2 (1.8)	2 (2.2)	0	
Reoperation	7 (6.3)	7 (7.6)	0	.348
Emergency	25 (22.3)	23 (25.0)	2 (10.0)	.235
BITA use	5 (4.5)	2 (2.2)	3 (15.0)	.039
Prosthetic grafft use	30 (26.8)	24 (26.1)	6 (30.0)	.782
Operative time	352.5 ± 126.8	343.8 ± 120.9	392.3 ± 148.2	.192
CPB time	183.7 ± 78.0	177.7 ± 71.2	215.3 ± 104.6	.231
AC time	132.0 ± 56.2	129.4 ± 55.3	147.2 ± 61.4	.320
Prolonged ventilation	32 (28.6)	28 (30.4)	4 (20.0)	.432
Sternal reexploration	8 (7.1)	6 (6.5)	2 (10.0)	.632
ICU stay	5.7 ± 6.9	5.9 ± 7.3	4.7 ± 4.3	.864
Operative mortality	13 (11.6)	10 (10.9)	3 (15.0)	.699
Fever elavation > 3 days	54 (48.2)	42 (45.7)	12 (60.0)	.325
Postive culture for SA	18 (16.1)	6 (6.5)	12 (60.0)	<.001
Postive culture for other organisms	17 (15.2)	15 (16.3)	2 (10.0)	0.732
Repeat bacteremia	11 (9.8)	3 (3.3)	8 (40.0)	<.001
WBC (×102/mm3)	122.2 ± 54.5	116.9 ± 51.9	146.2 ± 61.2	.030
CRP (mg/dl)	11.4 ± 8.3	10.2 ± 7.5	17.0 ± 10.0	.002

BMI, body mass index; NYHA, New York Heart Association; LVEF, left ventricular ejection fraction; SA, Staphylococcus aureus; BITA, bilateral internal thoracic arteries; AC time, aortic clamp time; CPB, cardiopulmonary bypass; WBC, white blood cell count; CRP, C-reacting protein.

**Figure 1:**
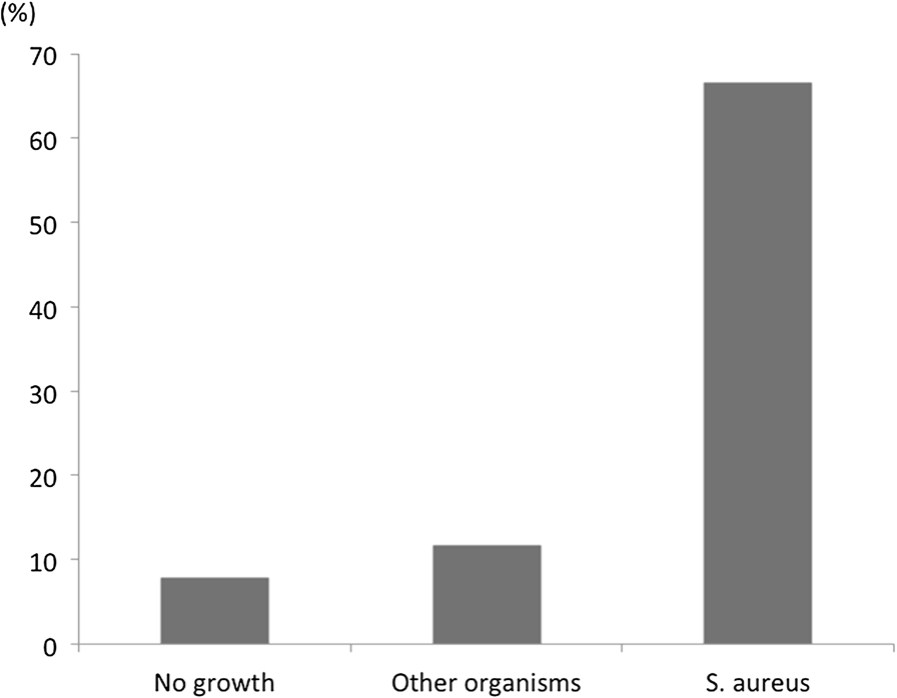
The prevalence of sternal wound infection by pathogen types (* indicates *p* **< 0.0001).**

Table 2 shows the culture results. Blood samples were cultured after a median of 13 postoperative days, and 35 cases (31.2%) had blood cultures that were positive for the presence of microorganisms, and 77 cases were negative. Blood samples were cultured on postoperative day 12 (2–90 days) in the group of patients who produced negative cultures, whereas blood culture was carried out on postoperative day 20 (6–61 days) in the positive group (*p* = 0.05). The organisms found in blood cultures are: *Staphylococcus aureus*, 18 cases (Methicillin- resistant species, 10 cases); Coagulase-negative *Staphylococcus*, 7 cases; and others, 8 cases. Sternal wound infection was observed in 22 cases (19.6%). The prevalence of sternal wound infection in patients whose cultures were negative culture and in those whose cultures were positive for other organism were 7.8% and 11.8%, respectively. However, 67% of patients whose cultures were positive for *Staphylococcus aureus* developed sternal infection (Figure 1, *p* < 0.0001). Of them, 8 patients required debridement of the infected tissues followed by reconstructive surgeries by omental and/or muscle flap, whereas others underwent only conservative managements such as negative pressure wound therapy. Preoperative characteristics and perioperative data were compared between patients with and without sternal wound infection (Table 1). The prevalence of hypertension and preoperative insulin use was significantly higher in the sternal wound infection group. The ratio of nasal carriage of *Staphylococcus aureus* was also higher in this group, but the difference was not statistically significant. There was no significant difference in types of operation, reoperation, emergency or prosthetic graft use between two groups. However, the use of internal thoracic artery was significantly higher in patients with sternal wound infection than those without. There was no significant difference in operative, cardiopulmonary bypass or aortic clamp time between the groups. Likewise, no difference was observed in the ratios of prolonged ventilation, sternal reexploration or operative mortality between the two groups. The prevalence of positive blood culture for *Staphylococcus aureus* was significantly higher in patients with sternal wound infection than those without infection, although no difference was observed in positive culture for other organisms between the 2 groups. Interestingly, there were significantly more patients with repeat bacteremia (positive blood cultures on at least 2 days) in the sternal wound infection group than that with no sternal infection. Postoperative white blood cell count and C-reacting protein levels were significantly higher in those with sternal infection than those without.

**Table 2:** Microbiology of blood culture

	**n (%)**
Negative	77 (68.8)
Positive	35 (31.2)
Staphylococcus aureus	18 (16.0)
Methicillin-sensitive	8 (7.1)
Methicillin-resistant	10 (8.9)
CNS	7 (6.3)
Enterococcus spp.	2 (1.8)
Escherichia coli	2 (1.8)
Bacillus spp.	2 (1.8)
Anaerobes	2 (1.8)
Others	2 (1.8)

CNS, Coagulase-negative *Staphylococcus.*

Multivariate analysis was performed to identify risk factors for sternal wound infection. It was demonstrated that the use of the bilateral internal thoracic arteries, blood culture positive for *Staphylococcus aureus*, and repeat bacteremia were statistically significant. Although preoperative insulin use was previously reported as a risk factor, the *p* value was not statistically significant, suggesting poor interaction with sternal wound infection of this factor in this particular series (Table 3).

**Table 3:** Multivariate analysis for sternal infection*

	**OR**	**95% CI**	**p value**
Insulin use	2.14	0.36-11.50	0.390
BITA use	13.68	1.09-167.36	0.043
Positive blood culture for SA	19.51	4.46-104.33	< 0.0001
Repeat bacteremia	17.98	2.51-161.77	0.004
WBC (×102/mm3)	0.99	0.984-1.014	0.913
CRP (mg/dl)	1.04	0.96-1.138	0.316

*Hypetension was excluded from the multivariate analysis because its estimates and conference intervals were not suitable for the multivariate logistic regression analysis.

## Discussion

Sternal wound infection is a rare and serious complication, and it is often difficult to make an early diagnosis because of frequent lack of clinical signs (i.e., pain, redness, swelling or purulent discharge) in the initial stages. Since bloodstream infection usually occurs immediately after a major primary infection, a positive blood culture is an important early sign of surgical site infection. Our results clearly demonstrated that blood stream infection by *Staphylococcus aureus* was a strong risk factor of sternal wound infection. This is consistent with previous reports, which showed that *Staphylococcus aureus* bacteremia is one of the most reliable tests for the association with sternal wound infection [9-12]. Another notable finding was that patients with repeat bacteremia are also likely to develop sternal wound infection. These findings suggest that our protocol of repeat sampling of blood culture in febrile patients provides important information on early diagnosis and treatment for sternal wound infections.

It has been widely documented that *Staphylococcus aureus* is the most frequent causative organism of sternal wound infection [12-14]. Sharma and colleagues reported that 49% of all cases of sternal wound infection after coronary bypass surgery were caused by *Staphylococcus aureus*, and one third involved methicillin-resistant strains [2]. Not only is *Staphylococcus aureus* the most common pathogen, it is also one of the most virulent organisms that cause serious systemic infections, and is strongly associated with a poor outcome [15,16]. Our routine strategy for febrile patients included immediate initiation of antibiotic regimens, focusing on gram-positive species, usually through the use of vancomycin or daptomycin depending on renal function [4,5]. These regimens are not associated with inadequate therapy nor increased cost [17]. Considering these findings, our initial treatment regimens seemed quite appropriate.

Blood culture contamination leads to unnecessary tests and treatments, and increases patient morbidity and medical expenditure [18]. Our protocol was designed to eliminate the possibility of false positive cultures as much as possible, as repeat positive cultures may interpret “true bacteremia” more precisely than a single positive culture [19]. In the present study, it was demonstrated that repeat bacteremia was a risk factor of sternal wound infection. In contrast, only 11% of patients with positive blood culture for organisms other than *Staphylococcus aureus* had sternal wound infection, suggesting the presence of other infectious sources or increased possibility of contamination. Therefore, it might be useful to obtain blood cultures multiple times to reduce contamination and subsequent nonessential treatments.

Whether nasal carriage of *Staphylococcus aureus* is associated with sternal wound infection after cardiac surgery is controversial [20-23]. Previously, Kluytmans and colleagues reported that nasal carriage of *Staphylococcus aureus* is a strong risk factor for sternal infection after cardiac surgery, and that intranasal mupirocin treatment decreased surgical site infection [20]. These findings were also supported by others [21]. In contrast, Perl and colleagues showed that there was no evidence of a strong relationship between nasal carriage of the organism and sternal infection [22]. Robiscek and colleagues demonstrated that mupirocin treatment did not reduce surgical site infection, but did reduce other systemic infections caused by methicillin-resistant *Staphylococcus aureus *[23]. In the present study, there was no association between the nasal carriage of *Staphylococcus aureus* and sternal wound infection. This might be, at least in part, due to preoperative mupirocin treatment for all nasal carriers of *Staphylococcus aureus*. It is yet to be determined whether preoperative nasal carriage plays a role in developing sternal wound infection.

Other controversial factors included diabetes managed by insulin administration, which some researchers have indicated as a risk factor for sternal wound infection [24,25] In the present study, preoperative insulin use was significantly higher in the group of patients with sternal wound infection. However, it failed to be confirmed as a risk factor for sternal wound infection by multivariate analysis. The difference between the present results and others might be attributed to the patient population and selection, as we did not include all afebrile patients who were at lower risk of systemic infection. Moreover, our perioperative intravenous insulin therapy was targeted at achieving a preprandial blood glucose level of 80 to 140 mg/dl, which is a stricter control than others [26]. This may explain some observed risk reduction in our patient cohort. Likewise, bilateral internal mammary artery use has raised controversy over the incidence of sternal wound infection [27,28]. In the present study, bilateral internal mammary artery use was another risk factor of sternal wound infection. With regard to internal mammary artery harvest, we have recently applied the “skeletonized technique” using an ultrasonic scalpel. It is expected that this technique may improve outcomes by decreasing the chance of sternal wound infection [29].

The retrospective nature of the study is a major limitation. This study reports a single institutional experience, the sample size was small and the follow-up period was short in some patients. As previously mentioned, we did not include afebrile patients in this study; this may have lead to selection bias. Propensity score matching was not possible due to the small numbers of patients in each group. Baseline etiology of cardiac disease and concomitant procedures were diverse, which might have affected the short- and long-term results. Although hypertension was a factor that was significant in univariate analysis, it was not included in multivariate logistic regression analysis because it caused the estimates and the conference intervals not stable in the analysis because only 1 patient of the “sternal infection present” group had hypertension. This exclusion did not affect the overall results or conclusions.

## Conclusions

Our routine protocol for extensive bacteriological studies including repeat blood culture studies on febrile patients was deemed quite useful for early detection of secondary bloodstream infection as well as a type of organism. *Staphylococcus aureus* and repeat bacteremia were associated with sternal would infection. Therefore, it is suggested that these blood culture results warranted expeditious radiological studies and aggressive surgical treatments. Nevertheless, it is of utmost importance to take precautions such as appropriate treatment of diabetes, stringent control of postoperative blood glucose level, appropriate prepping and draping the skin, and using double gloves and sufficient amount of irrigation in the operating room.

## Competing interests

In preparing the manuscript, the authors have no commercial association or sources of support that may pose competing interests.

## Authors’ contributions

TN conducted the study, and prepared all part of the manuscript. DT reviewed and performed statistical analyses. MN and HM helped with correcting the data and background literature review. YS helped with interpreting the data, emphasized the significance of this topic, and helped proofread the manuscript. All authors read and approved the final manuscript.

## References

[B1] Olbrecht VA Barreiro CJ Bonde PN Williams JA Baumgartner WA Gott VL Conte JV Clinical outcomes of noninfectious sternal dehiscence after median sternotomy Ann Thorac Surg 2006 9 902 10.1016/j.athoracsur.2006.04.058 16928505

[B2] Sharma M Berriel-Cass D Baran J Jr Sternal surgical-site infection following coronary artery bypass graft: prevalence, microbiology, and complications during a 42-month period Infect Control Hosp Epidemiol 2004 9 468 471 10.1086/502423 15242193

[B3] Tang GH Maganti M Weisel RD Borger MA Prevention and management of deep sternal wound infection Semin Thorac Cardiovasc Surg 2004 9 62 69 10.1053/j.semtcvs.2004.01.005 15366689

[B4] Popov AF Schmitto JD Jebran AF Bireta C Friedrich M Rajaruthnam D Coskun KO Braeuer A Hinz J Tirilomis T Schoendube FA Treatment of gram-positive deep sternal wound infections in cardiac surgery–experiences with daptomycin J Cardiothorac Surg 2011 9 112 10.1186/1749-8090-6-112 21929771 PMC3184046

[B5] Weis F Heyn J Hinske CL Vogt F Weis M Kur F Hagl C Beiras-Fernandez A Daptomycin as supportive treatment option in patients developing mediastinitis after open cardiac surgery J Cardiothorac Surg 2012 9 81 10.1186/1749-8090-7-81 22943887 PMC3485632

[B6] Kirkland KB Briggs JP Trivette SL Wilkinson WE Sexton DJ The impact of surgical-site infections in the 1990s: attributable mortality, excess length of hospitalization, and extra costs [see comments] Infect Control Hosp Epidemiol 1999 9 725 730 10.1086/501572 10580621

[B7] Managram AJ Horan TC Pearson ML Silver LC Jarvis WR The Hospital Infection Control Practices Advisory Committee. Guideline for prevention of surgical site infection 1999 Infect Control Hosp Epidemiol 1999 9 247 280 10.1086/501620 10219875

[B8] Garner JS Jarvis WR Emori TG Horan TC Hughes JM Olmsted RN CDC definitions for nosocomial infections APIC Infect Control App Epdemiol: Principles and practice 1996 St. Louis: Mosby A1 A20

[B9] Kanafani ZA Arduino JM Muhlbaier LH Kaye KS Allen KB Carmeli Y Corey GR Cosgrove SE Fraser TG Harris AD Karchmer AW Lautenbach E Rupp ME Peterson ED Straus WL Fowler VG Jr Incidence of and preoperative risk factors for Staphylococcus aureus bacteremia and chest wound infection after cardiac surgery Infect Control Hosp Epidemiol 2009 9 242 248 10.1086/596015 19199534

[B10] San Juan R Aguado JM López MJ Lumbreras C Enriquez F Sanz F Chaves F López-Medrano F Lizasoain M Rufilanchas JJ Accuracy of blood culture for early diagnosis of mediastinitis in febrile patients after cardiac surgery Eur J Clin Microbiol Infect Dis 2005 9 182 189 10.1007/s10096-005-1302-1 15776251

[B11] Fowler VG Jr Kaye KS Simel DL Cabell CH McClachlan D Smith PK Levin S Sexton DJ Reller LB Corey GR Oddone EZ Staphylococcus aureus bacteremia after median sternotomy: clinical utility of blood culture results in the identification of postoperative mediastinitis Circulation 2003 9 73 78 10.1161/01.CIR.0000079105.65762.DB 12821547

[B12] Chaudhuri A Shekar K Coulter C Post-operative deep sternal wound infections: making an early microbiological diagnosis Eur J Cardiothorac Surg 2012 9 1304 1308 10.1093/ejcts/ezr239 22241000

[B13] Gårdlund B Bitkover CY Vaage J Postoperative mediastinitis in cardiac surgery - microbiology and pathogenesis Eur J Cardiothorac Surg 2002 9 825 830 10.1016/S1010-7940(02)00084-2 12062270

[B14] Wertheim HF Melles DC Vos MC van Leeuwen W van Belkum A Verbrugh HA Nouwen JL The role of nasal carriage in Staphylococcus aureus infections Lancet Infect Dis 2005 9 751 762 10.1016/S1473-3099(05)70295-4 16310147

[B15] Olsen MA Krauss M Agniel D Schootman M Gentry CN Yan Y Damiano RJ Jr Fraser VJ Mortality associated with bloodstream infection after coronary artery bypass surgery Clin Infect Dis 2008 9 1537 1546 10.1086/587672 18419488

[B16] Chen LF Arduino JM Sheng S Muhlbaier LH Kanafani ZA Harris AD Fraser TG Allen K Corey GR Fowler VG Jr Epidemiology and outcome of major postoperative infections following cardiac surgery: risk factors and impact of pathogen type Am J Infect Control 2012 9 963 968 10.1016/j.ajic.2012.01.012 22609237 PMC3535474

[B17] Eagye KJ Kim A Laohavaleeson S Kuti JL Nicolau DP Surgical site infections: does inadequate antibiotic therapy affect patient outcomes? Surg Infect (Larchmt) 2009 9 323 331 10.1089/sur.2008.053 19622027

[B18] Waltzman ML Harper M Financial and clinical impact of false-positive blood culture results Clin Infect Dis 2001 9 296 299 10.1086/321881 11438892

[B19] Towns ML Jarvis WR Hsueh PR Guidelines on blood cultures J Microbiol Immunol Infect 2010 9 347 349 10.1016/S1684-1182(10)60054-0 20688297

[B20] Kluytmans JA Mouton JW VandenBergh MF Manders MJ Maat AP Wagenvoort JH Michel MF Verbrugh HA Reduction of surgical-site infections in cardiothoracic surgery by elimination of nasal carriage of Staphylococcus aureus Infect Control Hosp Epidemiol 1996 9 780 785 10.2307/30141170 8985763

[B21] Hebert C Robicsek A Decolonization therapy in infection control Curr Opin Infect Dis 2010 9 340 345 10.1097/QCO.0b013e32833ae214 20485164

[B22] Perl TM Cullen JJ Wenzel RP Zimmerman MB Pfaller MA Sheppard D Twombley J French PP Herwaldt LA Mupirocin And The Risk Of Staphylococcus Aureus Study Team. Intranasal mupirocin to prevent postoperative Staphylococcus aureus infections N Engl J Med 2002 9 1871 1877 10.1056/NEJMoa003069 12063371

[B23] Robicsek A Beaumont JL Thomson RB Jr Govindarajan G Peterson LR Topical therapy for methicillin-resistant Staphylococcus aureus colonization: impact on infection risk Infect Control Hosp Epidemiol 2009 9 623 632 10.1086/597550 19496730

[B24] Colombier S Kessler U Ferrari E von Segesser LK Berdajs DA Influence of deep sternal wound infection on long-term survival after cardiac surgery Med Sci Monit 2013 9 668 673 23942043 10.12659/MSM.889191 PMC3747019

[B25] Heilmann C Stahl R Schneider C Sukhodolya T Siepe M Olschewski M Beyersdorf F Wound complications after median sternotomy: a single-centre study Interact Cardiovasc Thorac Surg 2013 9 643 648 10.1093/icvts/ivs554 23355648 PMC3630417

[B26] Furnary AP Wu Y Eliminating the diabetic disadvantage: the Portland Diabetic Project Semin Thorac Cardiovasc Surg 2006 9 302 308 10.1053/j.semtcvs.2006.04.005 17395026

[B27] Diez C Koch D Kuss O Silber RE Friedrich I Boergermann J Risk factors for mediastinitis after cardiac surgery - a retrospective analysis of 1700 patients J Cardiothorac Surg 2007 9 23 10.1186/1749-8090-2-23 17511885 PMC1891287

[B28] Agrifoglio M Trezzi M Barili F Dainese L Cheema FH Topkara VK Ghislandi C Parolari A Polvani G Alamanni F Biglioli P Double vs single internal thoracic artery harvesting in diabetic patients: role in perioperative infection rate J Cardiothorac Surg 2008 9 35 10.1186/1749-8090-3-35 18573201 PMC2467417

[B29] Sá MP Ferraz PE Escobar RR Vasconcelos FP Ferraz AA Braile DM Lima RC Skeletonized versus pedicled internal thoracic artery and risk of sternal wound infection after coronary bypass surgery: meta-analysis and meta-regression of 4817 patients Interact Cardiovasc Thorac Surg 2013 9 849 857 10.1093/icvts/ivt012 23446674 PMC3653453

